# Body Mass Index Is Associated With Mucosal Disease in Crohn’s: Results of a Case-Control Study

**DOI:** 10.14740/gr631w

**Published:** 2014-12-27

**Authors:** Talha A. Malik, Richard A. Kaslow, Stacey S. Cofield, Peter J. Mannon

**Affiliations:** a Department of Medicine-Gastroenterology, University of Alabama at Birmingham, Birmingham, AL, USA; b Department of Epidemiology, University of Alabama at Birmingham, Birmingham, AL, USA; c Department of Biostatistics, University of Alabama at Birmingham, Birmingham, AL, USA

**Keywords:** Body mass index, Obesity, Crohn’s disease

## Abstract

**Background:**

Recent studies have suggested that increased body mass index (BMI) may have an adverse effect on treatment outcomes and natural history in Crohn’s disease (CD). We aimed to test the hypothesis that CD patients with higher BMI would be more likely than those with lower BMI to have persistent active mucosal disease.

**Methods:**

We designed a case-control study. Sample population comprised CD patients with active disease at the beginning of observation. At the end of observation, cases had persistent active mucosal disease and controls had entered remission. With multivariable logistic regression models, we evaluated the effect of baseline BMI as a continuous variable and a categorical variable on persistent active mucosal disease.

**Results:**

We analyzed data from 104 patients (36 cases and 68 controls). In a model containing BMI as a continuous variable, higher BMI was significantly associated with persistent active mucosal disease (odds ratio (OR) = 1.09 per unit increase; 95% confidence interval (CI), 1.02 - 1.17; P = 0.012). In a model containing BMI as a categorical variable, obese patients were 2.7 times more likely to have persistent active mucosal disease compared to non-obese patients (OR = 2.72; 95% CI, 1.00 - 7.35; P = 0.049).

**Conclusion:**

Excessive weight measured both quantitatively as BMI and categorically as obesity in CD patients is associated with persistent active mucosal disease.

## Introduction

Crohn’s disease (CD) is a chronic inflammatory condition, primarily of the intestinal tract that affects 750,000 people in the US. It is characterized by transmural inflammation and can involve any part of the gastrointestinal tract [1, 2]. Despite well-characterized genotypic and phenotypic characteristics of affected patients, CD responds variably and unpredictably to treatment [3-6].

Adipose tissue is an active endocrine organ that is made up of elements of connective tissue as well as cells represented by pre-adipocytes and adipocytes that can be prominent mediators of inflammation in the human body [6-9]. Body mass index (BMI), the most commonly used clinical measure of the degree of adipose tissue in the body is calculated by dividing weight (in kilograms) by the height (in meters) squared and a BMI of ≥ 30 kg/m^2^ is categorized as representing “obesity” [6]. In the past few decades, the prevalence of obesity throughout the US and the world has markedly increased [10]. This trend in obesity is reflected in patients with CD as well [11, 12]. Despite a rising number of obese CD patients, research on the influence of high BMI, especially obesity on objective outcomes in CD patients remains sparse. Data based on sound epidemiologic research are especially lacking when it comes to the now widely accepted disease outcome parameter in CD of mucosal (endoscopic) remission instead of mere clinical response [13, 14]. Study of the relationship between obesity and objective outcomes in CD is especially important because of credible molecular evidence that links adipose tissue physiology to intestinal inflammation. However, it is unclear whether this link translates into a causal or clinically meaningful association between obesity and CD.

Blain and colleagues found an association between obesity and rapid progression of disease (measured clinically) in CD patients [13]. In a subsequent study by Hass et al, overweight or obese CD patients experienced more rapid progression to first surgical intervention compared to underweight patients [14]. However, this difference in rate of progression of disease was not significantly different between obese and normal weight patients [14]. Drawing on our own observations and recent findings of others (not always consistent in regard to the association between rising BMI, especially the obese state and poor outcome), we aimed to test the hypothesis that CD patients with higher BMI (examined as a continuous variable as well as a categorical variable using obesity as reference) would be more likely than those with lower BMI to have persistently active mucosal (endoscopic) disease [13-16].

## Methods

### Study design, patient population, and selection criteria

Following approval by the University of Alabama’s Office of the Institutional Review Board, we began a retrospective unmatched case-control study of outpatients in the adult IBD clinic at the University of Alabama at Birmingham (UAB). Cases were patients 19 years or older who had 1) weight and height documented at the beginning of observation, 2) at least 1 year of follow-up, 3) an established diagnosis of active CD at the beginning of observation, and 4) persistent active mucosal ulceration upon evaluation by endoscopy at the end of observation (without regard to intervening state of clinical disease activity). Controls in our study were represented by CD patients seen in the same clinic as the cases. They met the first three criteria above but achieved mucosal remission at end of observation (without regard to intervening state of clinical disease activity). Patients who developed cancer or required emergent mucosal (endoscopic) evaluation during observation were excluded. Controls were selected by the method of cumulative sampling. Sampling was independent of the distribution of the exposure of interest, BMI.

### Data

For both cases and controls, information obtained from medical records included: 1) demographic data (age, ethnicity, gender, height and weight); 2) clinical data (age at diagnosis and end of observation, tobacco use, duration, location and behavior of CD as well as clinical and mucosal disease activity at the beginning and end of observation); and 3) medication history (use of steroids, traditional immune modulators and biological agents). Information on family history, extra-intestinal manifestations and diabetes mellitus was also collected. Data were recorded on a standardized data collection form and then entered into a database. All demographic data were abstracted from the medical record by one investigator and were reviewed randomly for accuracy by a second. Outcome data were collected by TM. Data abstractors were unaware of patient demographics including BMI.

Cases were defined as those CD patients who had persistent mucosal (endoscopic) inflammation as defined in the procedure report by the endoscopist. The language was standardized with the requirement that documentation of positive ulceration in the report be fulfilled. Histological confirmation was not required. Controls were defined as those CD patients who were in mucosal (endoscopic) remission as defined in the procedure report by the endoscopist. The language was standardized. Histological confirmation of histological disease inactivity was not required.

BMI was calculated as the ratio of weight to height squared documented in the medical record at initial observation. As already mentioned in the introduction, CD patients with a BMI ≥ 30 kg/m^2^ were categorized as obese. Those with a lower BMI were categorized as non-obese (≥ 25 and < 30 kg/m^2^ = overweight and ≥ 18 and < 25 kg/m^2^ = standard weight). BMI was assessed in two ways: 1) BMI as a continuous variable; 2) BMI as obese/non-obese. Covariates in both models were age at end of observation, ethnicity, and gender, duration of disease and observation period. In regard to location of CD, patients were divided into those with small bowel, ileocolonic or colonic disease according to the Montreal classification of CD. Patients were considered to have perianal disease if they had a history of perianal fistula or abscess. Patients with a previous history of anal stricture, anal fissure, hemorrhoid, skin tags or anal ulcer in the absence of a clearly documented history of perianal fistula or abscess were not considered to have perianal disease in this study. Treatment with corticosteroid medication was defined as use of an oral or parenteral corticosteroid agent for more than 1 week, of a rectal or topical agent for more than a month, or of oral budesonide for more than 3 months during the period of observation. Immune modulator treatment was defined as use of azathioprine (AZA), 6-mercaptopurine (6-MP), methotrexate (MTX) or any biological agent for at least 4 weeks during the period of observation. Eligibility for the study was not dependent on use or non-use of any of these agents. Distinction was not made among different biological agents. Similarly, data on cumulative dose and duration of medications were not collected.

Smoking was defined as use of tobacco products at any time during observation period. Current, ever and never smokers were not distinguished. We did not differentiate between cigarette, cigar, pipe or smokeless tobacco use.

### Statistical analysis

Demographic characteristics and clinical information were compared between cases and controls using Chi-square test or Fisher exact test for categorical variables and independent *t*-test for continuous variables. However, if evidence showed that the data did not follow a normal distribution, Wilcoxon rank sum test, a nonparametric analog of the *t* test, was used instead. Unadjusted odds ratios (ORs) and 95% confidence intervals (CIs) were estimated for the association between BMI as a continuous as well as categorical (obese vs. non-obese) variable and outcome of interest (persistent active mucosal disease) by logistic regression. We selected age, gender, ethnicity, duration of disease and duration of observation as covariates in adjusted models. We also arbitrarily decided to add any other covariate with a P value of < 0.1 for an unadjusted association between it and persistent active mucosal (endoscopic) disease. Finally, multivariable logistic regression analyses adjusted for age, gender, ethnicity, duration of disease and duration of observation were performed. The first adjusted model contained BMI as a continuous independent variable and the second one contained BMI dichotomized into obese vs. non-obese categories. In both models, the outcome of interest was active (cases) vs. inactive mucosal disease (controls). Results are presented as median (range) and OR (95% CI), unless noted. Statistical tests were two-sided with a 5% significance level (i.e., α = 0.05). Statistical software (SAS, version 9.2; SAS Institute, Inc., Cary, NC, USA) was used to perform all statistical analyses.

### Ethical statement

University of Alabama Office of Institutional Review Board approved this study.

## Results

**Figure 1:**
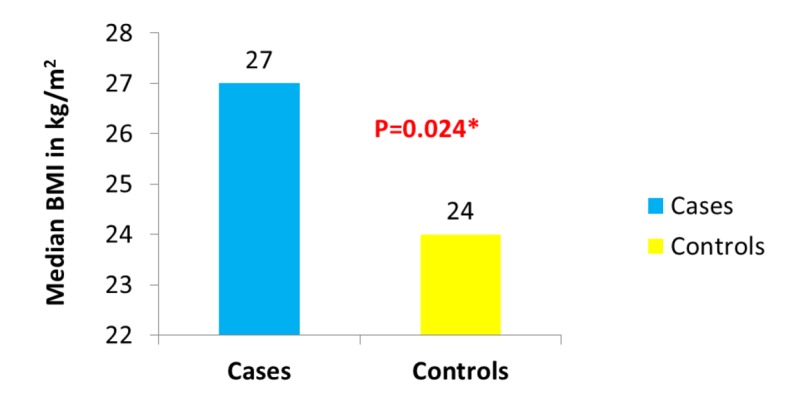
Distribution of BMI in cases vs. controls.

**Figure 2:**
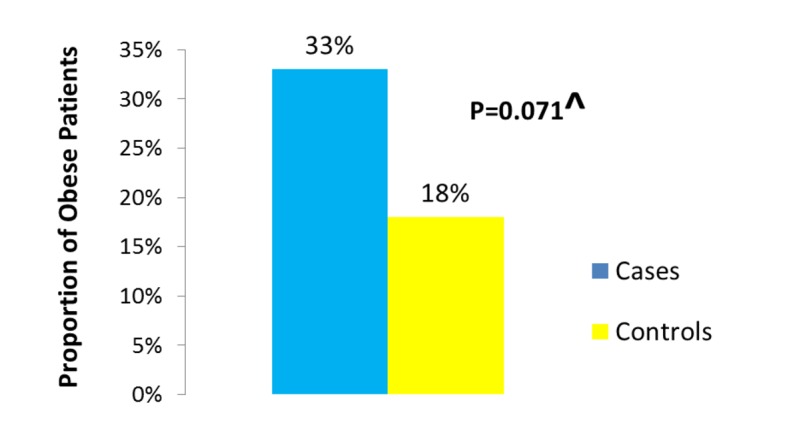
Proportion of obese among cases vs. controls.

Of the 1,449 patients seen at UAB for CD, we were able to identify 104 who fulfilled the stringent criteria of having their weight and height documented at the beginning of observation, having at least 1 year of follow-up, having clearly documented evidence of active mucosal (endoscopic) CD at the beginning of observation, and having a clearly documented state of mucosal (endoscopic) disease activity upon evaluation at the end of observation. Thirty-six of the 104 patients were cases as they had active mucosal (endoscopic) disease at the end of observation. Sixty-eight of the 104 patients were controls as they had achieved mucosal (endoscopic) remission at the end of observation. The median BMI was significantly different between cases (approximately 27 kg/m^2^) and controls (24 kg/m^2^) (P = 0.024) (Fig. 1). The proportion of obese patients was higher among cases compared to controls (33% of cases vs. 18% of the controls) (P = 0.071) (Fig. 2). No significant difference was detected between cases and controls in median age at the end of observation. A comparison of other characteristics of cases and controls appears in Table 1.

**Table 1:** Characteristics of Cases and Controls

Characteristic	Cases (N = 36)	Controls (N = 68)
BMI, median (range)	27 (19 - 44)	24 (19 - 46)
Obese	12 (33%)	12 (18%)
Age, median (range)	37 (20 - 79)	35 (19 - 72)
Duration of disease, median (range)	5 (1 - 37)	7 (1 - 36)
Duration of observation, median (range)	1 (1 - 4)	2 (1 - 4)
Caucasian/African American	18/18 (50%/50%)	26/42 (38%/62%)
Men/women	15/21 (42%/58%)	25/43 (37%/63%)
Age at diagnosis, median (range)	27 (10 - 75)	25 (11 - 68)
Perianal disease	12 (33%)	24 (35%)
Tobacco consumption	9 (25%)	13 (19%)
Steroid use	16 (44%)	20 (29%)
FH AID	6 (17%)	12 (18%)
FH cancer	10 (29%)	13 (20%)
FH CRC	3 (5%) (N = 35)	4 (11%) (N = 66)
FH IBD	5 (14%)	7 (10%)
AZA/6-MP use	19 (53%)	35 (51%)
Biologic use	25 (69%)	50 (73%)
Methotrexate use	6 (17%)	8 (12%)
Diabetes mellitus	4 (11%)	5 (7%)
EIM	18 (50%)	23 (34%)

A logistic regression model was applied to assess the association between BMI as a continuous variable and mucosal (endoscopic) disease activity; the model included age, gender, ethnicity, duration of disease and observation period as covariates (none of these nor any of the other covariates had a P value of < 0.1 for an unadjusted association between them and persistent active mucosal disease). This model fulfilled statistical criteria for a good fit supporting the likelihood that the model appropriately explained the variation in mucosal (endoscopic) disease activity in CD patients.

Having a higher BMI was associated in a significant manner with persistent active mucosal (endoscopic) disease (OR = 1.09; 95% CI, 1.02 - 1.17; P = 0.012). That is, each unit/point increase in BMI was associated with a 9% higher probability of having active mucosal (endoscopic) disease.

In a similarly appropriately fitted logistic regression model containing BMI as a categorical variable (obese vs. non-obese patients) along with age, gender, ethnicity, duration of disease and observation period as covariates, presence of persistent active mucosal (endoscopic) disease was likewise associated with being obese vs. non-obese (OR = 2.72; 95% CI, 1.00 - 7.35; P = 0.0492). The effect of the covariates including the exposures of interest appears in Table 2.

**Table 2:** Difference in Relevant Covariates Between Cases and Controls

Covariate	Cases (N = 36)	Controls (N = 68)	P value
BMI, median (range)	27 (19 - 44)	24 (19 - 46)	0.024
Obese	12 (33%)	12 (18%)	0.0709
Age, median (range)	37 (20 - 79)	35 (19 - 72)	0.413
Duration of disease, median (range)	5 (1 - 37)	7 (1 - 36)	0.1742
Duration of observation, median (range)	1 (1 - 4)	2 (1 - 4)	0.69
Caucasian/African American	18/18 (50%/50%)	26/42 (38%/62%)	0.249
Men/women	15/21 (42%/58%)	25/43 (37%/63%)	0.625

The full multivariable models for BMI as a categorical and a continuous variable appear in Tables 3 and 4 respectively.

**Table 3:** Multivariable Full Model for BMI as a Categorical Variable*

Effect	Point estimate	95% CI	P value
Obese vs. non-obese	2.716	1.004 - 7.349	0.0492
Age now	0.994	0.962 - 1.027	0.7065
Sex	1.567	0.627 - 3.913	0.3361
Race	1.748	0.725 - 4.215	0.2135
Duration of observation	0.824	0.505 - 1.325	0.4395
Duration of disease	0.995	0.941 - 1.052	0.8604

*Adjusted for age, sex, race, duration of disease and observation.

**Table 4:** Multivariable Full Model for BMI as a Continuous Variable*

Effect	Point estimate	95% CI	P value
BMI	1.09	1.019 - 1.166	0.0122
Age	0.992	0.959 - 1.025	0.6206
Sex	1.371	0.556 - 3.381	0.4933
Race	1.567	0.646 - 3.798	0.3206
Duration of observation	0.787	0.477 - 1.298	0.3486
Duration of disease	1.001	0.945 - 1.059	0.9828

*Adjusted for age, sex, race, duration of disease and observation.

## Discussion

Our case-control study shows that excessive weight measured both quantitatively as BMI and categorically as obesity is associated with higher rates of active mucosal (endoscopic) disease among CD patients in our IBD clinic.

Our findings are consistent with some but not all previous observational research reports on obesity and CD [13-16]. For example, Blain et al compared the clinical course of CD patients who were obese with those CD patients who were not obese [13]. They defined obesity in CD patients as a BMI greater than 25 kg/m^2^ at disease onset and more than 30 kg/m^2^ at any time during their subsequent disease course. Their obese CD patients were more likely to develop perianal disease when compared to non-obese CD patients. After adjusting for age, gender, disease duration as well as location of CD, their study also found that the length of time to development of perianal disease was shorter in obese CD patients. In their adjusted analysis, a higher proportion of obese vs. non-obese CD patients had disease flares and hospitalizations. However, no difference was detected between obese and non-obese CD patients based on long-term outcome, including outcome after excisional surgery [13].

Another group led by Hass categorized overweight and obese CD patients together into one group, i.e. all those with a BMI greater than 25 kg/m^2^, and compared them to CD patients with a normal BMI (less than 25 kg/m^2^) [14]. Within the latter group, the subset with a BMI of less than 18.5 kg/m^2^ was analyzed separately. Hass et al reported that patients with a BMI < 25 kg/m^2^ were older at diagnosis than the overweight or obese CD patients. However, in contrast to the study by Blain et al, they found no difference in the number of surgeries, rate of perianal disease or the degree of required therapy escalation between the two obese CD groups. Of note, Blain et al had compared obese to non-obese patients whereas Hass et al had combined overweight and obese patients into a single group. Interestingly, the latter investigators did find that overweight/obese patients experienced a shorter time to first surgical intervention than the subset that had a BMI of less than 18.5 kg/m^2^.

Retrospective observational design, relatively small sample size and consequently, selection bias were among important limitations of our study. We believe confounding by indication (for treatment) was not really an issue in this study as the rates of traditional and biological immune modulators were similar among cases and controls. Nevertheless, collecting meticulous data on type, dose and duration of traditional and biological immune modulator, preferably in the context of a prospective study may shed more light on these important effects. On account of the retrospective observational design of this study, we were not able to ensure documentation of mucosal (endoscopic) disease activity independent of clinical status. As earlier alluded to, we selected controls for our cases in this study by cumulative sampling instead of incident density (concurrent) sampling. Cumulative sampling identifies controls at the end of observation/study period. This is in contrast to incidence density sampling (controls identified at the same time as cases) as well as case-cohort sampling (controls identified at the beginning of observation period). We selected controls for our cases in this study by cumulative sampling because of the retrospective nature of our study and on account of stringent inclusion criteria. This led to selection bias but at the same time decreased information bias. Of note however, masking the data abstractors helped decrease some of the selection bias that without doubt occurred. To decrease information bias, we relied almost solely on medical record documentation verified by a second data abstractor for both components of BMI (height and weight) as well data on other covariates.

Corticosteroid use was not included into the multivariable models despite the observation that its use was numerically different between cases and controls (P = 0.125) because the association was not significant based on our cutoff P < 0.1. To examine its effect, additional sub-group analyses were performed in which steroid use was added to the adjusted models. Inclusion of corticosteroid use in the adjusted models gave the same P value for BMI (adjusted P = 0.01) and a slightly higher value for obesity (P = 0.06 from P = 0.05 with OR going down to 2.5 from 2.7). But with 36 outcomes of interest, adding additional covariates led to over-fitting; therefore we limited our adjusted models to our covariates of clinical interest. Special care was taken to include BMI/obesity data that were collected at the beginning of observation whereas steroid use was defined as “steroid use during observation”. Therefore steroid use (as defined in this study) was unlikely to have causally impacted BMI/obesity (as defined in this study). Also, based on the lack of statistically significant relationships between covariates of interest in our univariate analyses, we believe that our decision not to match cases with controls according to those characteristics probably did not lead to a substantial loss of statistical efficiency and did not appear to leave the findings vulnerable to significant confounding. Moreover, as already alluded to, use of stringent inclusion criteria for the sample increased validity of our findings despite lowering the eventual size of the sample. For example, care was taken when defining inclusion criteria in regard to length of observation as “at least 1 year of observation” and not “1 year of observation”. The range of observation for both cases and controls was 1 - 4 years with median of 1 year (rounded down to 1 year) among cases and 2 years among controls. Duration of observation was controlled for in the multivariable analysis (in order to account for any potential confounding due to it). We had reason to believe that those who underwent colonoscopy at the end of observation period were not necessarily very different from those who did not based on the outcome of interest, as in a tertiary care IBD center such as ours, IBDologists routinely perform colonoscopies or imaging studies to confirm mucosal (endoscopic) healing. The use of mucosal (endoscopic) disease activity rather than clinical disease activity as an outcome further increased internal validity of our findings in terms of a credible association between BMI and truly active Crohn’s in this sample.

The current study strengthens the hypothesis of a detrimental effect of BMI, specifically obesity, on the course of CD, specifically persistently active mucosal (endoscopic) inflammation. Understanding how the association of obesity with poorer Crohn’s outcomes contributes to its causality will require a richer understanding of the biological as well as the social and environmental factors that drive the pathogenesis, treatment options and ultimately, the course of CD. It is important to understand that obesity is as much a clinical construct as it is a molecular phenomenon as every obese person does not have metabolic syndrome. Therefore, initial consideration of the clinical context of obesity and CD association is critical to our generation of future hypotheses.

Future directions include larger prospective studies that allow capture of a wider range of important data elements by inclusion of more covariates into adjusted models to better account for potential confounding, and to test for association of other measures of adipokine-mediated mesenteric inflammation (such as mesenteric fat content and/or biomarkers) with parameters of outcome in CD.

## Abbreviations

CD: Crohn’s disease
BMI: body mass index
TNF-alpha: tumor necrosis factor alpha
AZA: azathioprine
6-MP: 6-mercaptopurine
MTX: methotrexate
SAS: statistical analysis software
